# Effects of simultaneous supplementation of 3-phytase and 6-phytase on phosphorus and calcium digestibility in growing pigs

**DOI:** 10.5713/ab.250775

**Published:** 2025-11-14

**Authors:** Hyunseok Do, Beob Gyun Kim

**Affiliations:** 1 Department of Animal Science, Konkuk University, Seoul, Korea

**Keywords:** Digestibility, Phosphorus, Phytase, Swine

## Abstract

**Objective:**

The present study aimed to determine the effects of supplemental 3-phytase, 6-phytase, or both on total tract digestibility of phosphorus (P) and calcium in corn-soybean meal-based diets fed to growing pigs.

**Methods:**

Twelve barrows with an initial body weight of 63.1±3.0 kg were allotted to a triplicated 4×3 incomplete Latin square design with 4 diets and 3 periods to obtain 9 observations per diet. The experimental diets comprised 1) a control diet based on 73.3% corn and 23.0% soybean meal without inorganic P source or supplemental phytase, 2) the control diet supplemented with 1,000 phytase unit (FTU)/kg of 3-phytase, 3) the control diet supplemented with 1,000 FTU/kg of 6-phytase, and 4) the control diet supplemented with both 3-phytase at 500 FTU/kg and 6-phytase at 500 FTU/kg.

**Results:**

Supplemental phytase reduced (p<0.05) the daily P output from pigs regardless of the phytase source or the combination of the 2 phytase sources. The pigs fed the diet supplemented with 6-phytase or both 3-phytase and 6-phytase had less daily P output compared with those fed the diet supplemented with only 3-phytase (p<0.05). The total tract digestibility of P in pigs fed the diet supplemented with both 3-phytase and 6-phytase was greater (p<0.05) than those fed the control diet or the diet supplemented with only 3-phytase. The apparent total tract digestibility of calcium in pigs fed the diet supplemented with 6-phytase or both 3-phytase and 6-phytase was greater compared with the control group (p<0.05).

**Conclusion:**

Taken together, supplemental phytase increased P digestibility in corn-soybean meal-based diets fed to growing pigs regardless of the phytase sources and the effects of supplemental phytase on the P and calcium digestibility were most pronounced in the mixture of 3-phytase and 6-phytase.

## INTRODUCTION

Phosphorus (P) is a mineral that plays an important role in the development and maintenance of the skeletal system [1,2]. Therefore, an adequate supply of P is essential for pigs. In plant feedstuffs including corn and soybean meal, a large proportion of P is poorly available to pigs due to the lack of endogenous phytase that can hydrolyze phytate in the ingredients fed to pigs [2–4]. Phytate exists as myo-inositol 1,2,3,4,5,6-hexakisphosphate complexed with various cations, proteins, and carbohydrates [1]. To enhance the utilization of P, exogenous phytase has been widely applied in the swine feeds [2]. Although the exogenous phytase dramatically increases P digestibility in pigs, standardized total tract digestibility (STTD) of P in most plant ingredients generally ranged from 58.0% to 70.0% even with approximately 1,000 phytase unit (FTU)/kg of exogenous phytase in pigs [5–7]. This indicates that a high dose of exogenous phytase may not fully hydrolyze phytate in the plant ingredients and a large quantity of P is still unavailable to pigs.

Phytase is classified as 3-phytase and 6-phytase based on the position of phosphate ester group in the phytate molecule where hydrolysis is initiated [8]. In broilers, the effects of simultaneous use of 3-phytase and 6-phytase have been studied to investigate a synergistic effect of the 2 phytase sources [9,10]. To our knowledge, however, no research has been documented on the effects of simultaneous use of 3-phytase and 6-phytase in pigs. Therefore, the objective of the present study was to determine the effects of supplementing 3-phytase, 6-phytase, or both to a corn-soybean meal-based diet on total tract digestibility of P in growing pigs.

## MATERIALS AND METHODS

### Animals, experimental design, and diets

Twelve barrows (Landrace×Yorkshire) with an initial body weight of 63.1±3.0 kg were allotted to a triplicated 4×3 incomplete Latin square design with 4 diets and 3 periods per square using a spreadsheet method to minimize potential carryover effects [11]. Pigs were individually housed in metabolic crates equipped with a feeder. The experimental diets comprised 1) a control diet based on 73.3% corn and 23.0% soybean meal without inorganic P source, 2) the control diet supplemented with 1,000 FTU/kg of 3-phytase, 3) the control diet supplemented with 1,000 FTU/kg of 6-phytase, and 4) the control diet supplemented with 500 FTU/kg of 3-phytase and 500 FTU/kg of 6-phytase (Table 1). The 3-phytase from *Aspergillus niger* (Granular Phytase; Anhui Zhengliang Kyushu Biotechnology) and 6-phytase from *Trichoderma reesei* (Axtra PHY GOLD 5 T; Danisco Animal Nutrition) contained approximately 10,000 and 5,000 FTU/g of phytase activity, respectively. One FTU corresponds to the quantity of phytase that liberates 1 μmol of inorganic P per minute from an excess of 15 *M* sodium phytate at pH 5.5°C and 37°C [12]. The thermostability of 3-phytase and 6-phytase was resistant up to 95°C and 80°C, respectively, based on the manufacturers’ information.

### Feeding and sample collection

The daily feed allowance per pig was calculated as 3.0 times the metabolizable energy requirements for maintenance (i.e., 197 kcal of metabolizable energy per kg of body weight^0.60^; NRC [1]) based on the initial body weight of the pigs in each period and the metabolizable energy of the experimental diets. The amount of feed allowance was divided into 2 equal quantities and provided to pigs at 08:00 and 17:00 h. The experimental diets were provided in mash form. Pigs had free access to water. Each period consisted of a 5-d adaptation period and a 5-d collection period and the marker-to-marker procedure was used for the quantitative collection of feces [13]. Chromic oxide was used as an indigestible marker for the initiation and termination of fecal collection. Fecal collection started when the marker began to appear in the feces and ended when the marker appeared again. All fecal samples were stored at −20°C immediately after collection.

### Chemical analyses

The fecal samples were dried in a forced-air drying oven at 55°C until constant weight was achieved and finely ground for chemical analyses. Experimental diets and fecal samples were analyzed for dry matter (method 930.15) and ash (method 942.05; AOAC [14]). The nitrogen concentrations of experimental diets and fecal samples were analyzed by the combustion method (method 999.03) using a Rapid N cube (Elementar Americas) with aspartic acid as the internal standard. Amylase-treated neutral detergent fiber (method 2002.04) and acid detergent fiber (method 973.18) of experimental diets were analyzed according to the procedures described in the AOAC [14]. Gross energy of experimental diets and feces was determined using bomb calorimetry (Parr 6400; Parr Instruments). The calcium (Ca) concentrations in the diets and feces were analyzed using atomic absorption spectrophotometer (Savant AA; GBC Scientific Equipment) based on the method 985.35 in the AOAC [14]. The P concentrations in the diets and feces were analyzed using an inductively coupled plasma spectroscopy (Optima 8300; PerkinElmer) based on the method 964.06 in the AOAC [14].

### Calculations and statistical analyses

The apparent total tract digestibility (ATTD) and STTD of P were calculated using the following equations:


(1) $$\text{ATTD of P }(\%)=({\text{P}}_{\text{intake}}-{\text{P}}_{\text{feces}})÷{\text{P}}_{\text{intake}}\times 100$$

(2) $$\text{STTD of P }(\%)=({\text{P}}_{\text{intake}}-[{\text{P}}_{\text{feces}}-\text{basal endogenous P losses}])÷{\text{P}}_{\text{intake}}\times 100$$

where P_intake_ and P_feces_ represent the amount of P intake (g/d) and fecal P output (g/d), respectively. The daily basal endogenous P losses (g/d) were calculated using the basal endogenous P losses of 190 mg/kg dry matter intake [1]. The ATTD of gross energy, dry matter, organic matter, crude protein, and Ca are also calculated.

Experimental data were analyzed using the MIXED procedures of SAS (SAS Institute). All values were within 1.5 times the interquartile range from the 1st or 3rd quartiles, and thus, no outlier was detected. The statistical model included diet as the fixed variable and replication, animal within replication, and period within replication as the random variables. Least square means were calculated, and the means were separated using the PDIFF option with Tukey’s adjustment. An individual pig was the experimental unit, and statistical significance was declared at p<0.05.

## RESULTS

Supplemental phytase reduced (p<0.05; Table 2) the daily P output from pigs regardless of the phytase source or the combination compared to the control diet. The pigs fed a diet supplemented with 6-phytase or both 3-phytase and 6-phytase had less daily P output compared with those fed a diet supplemented with 3-phytase (p<0.05). The total tract digestibility of P in pigs fed the diet supplemented with both 3-phytase and 6-phytase was greater than in those fed the control diet or the diet supplemented with 3-phytase (p<0.05). The ATTD of Ca in pigs fed the diet supplemented with 6-phytase or both 3-phytase and 6-phytase was greater compared with the control group (p<0.05). Supplemental phytase did not affect ATTD of gross energy, dry matter, organic matter, or crude protein.

## DISCUSSION

Phytases can be classified as 3-phytase or 6-phytase, depending on the position of the phosphate ester group on the phytate molecule where the hydrolysis is initiated [8,15]. The 3-phytase hydrolyzes the ester bond from the carbon-3 position whereas the 6-phytase initiates the hydrolysis at the carbon-6 position [16]. Compared with 3-phytase, 6-phytase is known to be more effective in degrading the bonds in phytates [17], leading to the formation of inositol phosphate isomers with lower binding affinity to proteins and minerals, and thereby allowing for a more efficient breakdown of phytate into lower inositol phosphates [18]. In other experiments, however, the effects of supplemental 6-phytase on P digestibility did not differ from those of 3-phytase in pigs [19,20], which is consistent with the present results.

A simultaneous use of different phytase products can be one of the approaches to maximize P digestibility by removing the phosphate groups from different positions on the inositol ring, thereby contributing to a more complete breakdown of the phytate molecule [21]. The simultaneous use of 3-phytase and 6-phytase can have a synergistic effect on P digestibility because 6-phytases have a strong preference for equatorial phosphate groups but are unable to cleave the axial phosphate group, and 3-phytase may cleave the axial phosphate group at the 3-carbon position [22,23]. In addition, variations in dephosphorylation pathways due to the phytase product can influence the profiles of lower inositol phosphate intermediates [9] which retain anti-nutritional properties of chelating minerals and reducing nutrient digestibility [18]. Therefore, we hypothesized that the simultaneous use of 3-phytase and 6-phytase would enhance the extent of phytate degradation and reduce the accumulation of lower inositol phosphate intermediates by efficiently hydrolyzing the axial phosphate group as well as the equatorial phosphate group, resulting in increased P and other nutrient digestibility.

The ATTD of Ca in the control group is similar to the observations in previous studies [4,24], which fed corn-soybean meal-based diets without inorganic P sources to pigs. Although the effects of phytase supplementation on the digestibility of energy and amino acids have been controversial, the increased digestibility of Ca by supplemental phytase has been consistent in previous experiments [4,16,24,25]. The increased ATTD of Ca by phytase can be explained by the breakdown of Ca–phytate complexes when the exogenous phytase is supplemented in pig diets [24]. Ingested Ca potentially binds with the phytate in the gastrointestinal tract of pigs, forming insoluble Ca-phytate complexes [26]. The exogenous phytase hydrolyzes the Ca-phytate complexes, thereby making Ca more available for absorption [24].

The ATTD of P in the control group is within the range reported in the literature, which fed corn-soybean meal-based diets without inorganic P sources to pigs [3,24,25]. The positive effects of supplemental 3-phytase have been observed in multiple experiments [19,27,28]. The 16.2 percentage unit increase in the ATTD of P by supplemental 3-phytase at 1,000 FTU/kg observed in the present experiment is consistent with the findings of Sands et al [28] who found a 13.7 percentage unit increase in the ATTD of P by supplementing a corn-soybean meal-based control diet with 1,200 FTU/kg of 3-phytase derived from *Aspergillus niger*. The positive effects of supplemental 6-phytase have been observed in previous experiments [4,16,25]. The effects of 6-phytase on the ATTD of P observed in the present experiment were comparable to the responses reported by Adedokun et al [16] who tested 1,000 FTU/kg of 6-phytase derived from *Trichoderma reesei*.

In the present study, the P digestibility in pigs fed the diet supplemented with both 3-phytase and 6-phytase was not different from that in pigs fed the diet supplemented with only 6-phytase. This finding is consistent with previous broiler studies [9,10] in which the effects of simultaneous supplementation of 3-phytase and 6-phytase on ileal P digestibility were comparable to the groups of 6-phytase only. The reason for the lack of increase in P digestibility by the combination of 3-phytase and 6-phytase compared with 6-phytase alone remains unclear. However, the present observations indicate that 6-phytase can be partially replaced with 3-phytase making a combination of phytase sources to save feed production cost. The production cost for 6-phytase is greater than 3-phytase [29]. Ennis et al [9] also suggested that phytase combinations at lower doses can perform similarly to higher single-source doses in poultry diets. Further research on varying ratios and doses of 3-phytase and 6-phytase is warranted to determine the substitution threshold at which 3-phytase can effectively replace 6-phytase without affecting P digestibility in pigs.

## CONCLUSION

Taken together, supplemental phytase increased P digestibility in corn-soybean meal-based diets fed to growing pigs, regardless of the phytase sources. The effects on the P and Ca digestibility of supplemental phytase were most pronounced in the mixture of 3-phytase and 6-phytase and was comparable to 6-phytase alone.

## Figures and Tables

**Table 1:** Ingredient and chemical composition of experimental diets (as-fed basis)

Item	Control diet	3-Phytase	6-Phytase	Combination^1)^
Ingredient (%)				
Ground corn	73.34	73.34	73.34	73.34
Soybean meal (45% crude protein)	23.00	23.00	23.00	23.00
_L_-Lys·HCl (78.8%)	0.14	0.14	0.14	0.14
Soybean oil	1.50	1.50	1.50	1.50
Ground limestone	1.40	1.40	1.40	1.40
Sodium chloride	0.30	0.30	0.30	0.30
Vitamin-mineral premix^2)^	0.30	0.30	0.30	0.30
Cornstarch	0.020	0.010	-	0.005
3-Phytase^3)^	-	0.010	-	0.005
6-Phytase^4)^	-	-	0.020	0.010
Calculated composition				
Metabolizable energy (kcal/kg)	3,383	3,383	3,382	3,382
Total phosphorus (%)	0.32	0.32	0.32	0.32
Total calcium (%)	0.59	0.59	0.59	0.59
Phytate-phosphorus (%)	0.24	0.24	0.24	0.24
Analyzed composition				
Dry matter (%)	88.6	88.5	88.9	88.6
Gross energy (kcal/kg)	3,953	3,946	3,955	3,930
Crude protein (%)	14.1	14.4	15.2	14.7
Ether extract (%)	5.0	5.0	4.7	4.7
Ash (%)	4.2	4.2	4.2	4.1
Amylase-treated neutral detergent fiber (%)	10.2	10.1	10.1	9.3
Acid detergent fiber (%)	4.1	4.0	4.0	4.0
Total calcium (%)	0.64	0.74	0.75	0.82
Total phosphorus (%)	0.25	0.27	0.24	0.26

1) A mixture of 3-phytase at 500 phytase unit (FTU)/kg and 6-phytase at 500 FTU/kg.

2) Provided the following quantities per kilogram of complete diet: vitamin A as retinyl acetate, 18,000 IU; vitamin D_3_ as cholecalciferol, 3,600 IU; vitamin E as _DL_-α-tocopheryl acetate, 60 mg; vitamin K as menadione nicotinamide bisulfite, 4.5 mg; thiamin as thiamine mononitrate, 4.5 mg; riboflavin, 7.5 mg; pyridoxine as pyridoxine hydrochloride, 4.5 mg; vitamin B_12_, 0.06 mg; _D_-pantothenic acid as _D_-calcium pantothenate, 30 mg; folic acid, 1.5 mg; niacin as nicotinamide, 45 mg; biotin, 0.3 mg; Co as cobaltous carbonate, 0.75 mg; Cu as copper sulfate, 120 mg; Fe as iron sulfate, 120 mg; I as calcium iodate, 0.75 mg; Mg as magnesium oxide, 60 mg; Mn as manganese sulfate, 60 mg; Se as sodium selenite, 0.3 mg; and Zn as zinc sulfate, 90 mg.

3) The 3-phytase product from *Aspergillus niger* (Granular Phytase; Anhui Zhengliang Kyushu Biotechnology) contained 10,000 FTU/g of phytase activity.

4) The 6-phytase product from *Trichoderma reesei* (Axtra PHY GOLD 5 T; Danisco Animal Nutrition) contained 5,000 FTU/g of phytase activity.

**Table 2:** Effects of supplemental 3-phyase, 6-phytase, or the combination of both phytase on total tract digestibility of phosphorus (P) and nutrients in pigs fed the corn-soybean meal-based diets

Item	Control	3-Phytase	6-Phytase	Combination	SEM	p-value
Intake (g/d)						
Feed intake	2,293	2,293	2,301	2,295	54	0.822
P intake	5.81^b^	6.08^a^	5.57^c^	6.04^a^	0.14	<0.001
Output						
Fecal output (g/d)	217	219	222	220	8	0.921
P in feces (%)	1.49^a^	1.09^b^	0.85^c^	0.88^c^	0.04	<0.001
P output (g/d)	3.21^a^	2.36^b^	1.90^c^	1.94^c^	0.11	<0.001
Apparent total tract digestibility (%)						
Gross energy	88.5	88.2	88.0	88.1	0.4	0.686
Dry matter	89.8	89.7	89.7	89.7	0.3	0.976
Organic matter	91.0	90.7	90.6	90.7	0.3	0.716
Crude protein	86.6	86.6	87.5	87.3	0.5	0.127
Calcium	61.0^b^	68.2^ab^	70.5^a^	72.7^a^	2.2	0.001
P	44.7^c^	60.9^b^	65.9^ab^	67.8^a^	1.9	<0.001
STTD of P^1)^ (%)	51.3^c^	67.2^b^	72.9^ab^	74.1^a^	1.9	<0.001

Each least square mean represents 9 observations.

The 3-phytase product from *Aspergillus niger* (Granular Phytase; Anhui Zhengliang Kyushu Biotechnology) contained 10,000 phytase unit (FTU)/g of phytase activity and the supplemental phytase at 0.01% provided 1,000 FTU/kg in the experimental diet. The 6-phytase product from *Trichoderma reesei* (Axtra PHY GOLD 5 T; Danisco Animal Nutrition) contained 5,000 FTU/g of phytase activity and the supplemental phytase at 0.02% provided 1,000 FTU/kg in the experimental diet. The combination represents a mixture of 3-phytase at 500 FTU/kg and 6-phytase at 500 FTU/kg.

1) The STTD of P was calculated using basal endogenous P losses of 190 mg/kg dry matter intake [1].

SEM, standard error of the mean; STTD, standardized total tract digestibility.

## Data Availability

Upon reasonable request, the datasets of this study can be available from the corresponding author.
